# Feed intake patterns of modern genetics lactating sows: characterization and effect of the reproductive parameters

**DOI:** 10.1186/s40813-022-00300-y

**Published:** 2023-01-31

**Authors:** María Rodríguez, Gonzalo Díaz-Amor, Joaquín Morales, Yuzo Koketsu, Carlos Piñeiro

**Affiliations:** 1 PigCHAMP Pro Europa S.L, C/Dámaso Alonso 14, 40006 Segovia, Spain; 2 School of Agriculture, Meiji University, Higashi-mita 1-1-1, Tama-ku, 214- 8571 Kawasaki, Kanagawa Japan

**Keywords:** Commercial swine herds, Feed consumption, Feeding behavior, Machine learning, Lactation, Reproductive performance

## Abstract

**Background:**

Knowing the feed intake pattern during lactation of modern genetic sows is crucial because it allows to anticipate possible problems and maximize their performance. On the other side, electronic feeders permit real-time data to be available for a more accurate evaluation of sow eating behavior. This work aimed to characterize the feed intake patterns of lactating highly prolific sows and determine their effect on reproductive performance. A database of 1,058 registers of feed intake collected from a commercial farm was used to identify five consistent sets of clusters (feeding curves) using machine learning. In the second step, the five feeding curves were characterized into five patterns by high, medium and low feed intake during 0–6 d and 7–28 d of lactation: 1-HH, 2-MH, 3-HM, 4-MM and 5-LL.

**Results:**

The mean daily feed intake of all the sows was 6.2 kg (0.06 SEM) across the 5 patterns. As the pattern numbers increased from 1-HH, 2-MH, 3-HM and 4-MM to 5-LL, their mean daily feed intake decreased from 7.6 to 6.9, 6.4, 5.8 and 4.3 (0.06 SEM) kg, respectively (*P* < 0.01). Sows with Pattern 1-HH tended to have shorter weaning-to-first service interval (*P* = 0.06) and had a higher farrowing rate than those with Pattern 5-LL (*P* < 0.01). Furthermore, contrast analysis showed that sows with Patterns 1-HH and 2-MH tended to have more piglets weaned (*P* = 0.05) and lower preweaning mortality (*P* = 0.07) than those with Patterns 3-HM and 4-MM. Also, sows with Patterns 1-HH and 3-HM had fewer stillborn piglets and a lower percentage of stillborn piglets and mummies than those with Patterns 2-MH and 4-MM (*P* < 0.01).

**Conclusions:**

This study indicates the importance of reaching Pattern 1-HH by rapidly increasing feed intake during early lactation and high feed intake during late lactation, which is associated with high weaning performance and subsequent reproductive performance of the sows. Also, the current study suggests that Pattern 1-HH is linked to good farrowing with a low percentage of stillborn piglets and mummies. Finally, it is critical for producers to timely identify a problem of sows’ eating behavior and to make a prompt decision to intervene.

## Background

Genetic selection over the last years for increased sow productivity, including litter weaning weight and the number of weaned pigs, has increased the demand for milk production [1, 2]. However, the lactational feed intake of sows has not increased to the same extent as the increased demand for milk production [3, 4]. In addition, as some studies have demonstrated, it is important to take into account the age of the animals because dietary requirements are different between multiparous and primiparous sows and that has a direct impact on their feed intake [5, 6].

For years, it has been well-documented in literature that insufficient nutrient intake in sows during lactation has serious reproductive and productive consequences [7]. Therefore, it is conceivable that increasing feed intake in lactating sows could reduce body weight losses, allow maintenance of body mass, and have less impact on production parameters [3, 8, 9]. These studies related to feed intake evaluation have been relatively easy to conduct in universities or small research units where only a small number of replicates are involved. Nevertheless, things get complicated when these studies are transferred to commercial farms, as they involve a significant monetary outlay and usually involve many personnel. Furthermore, these studies generally only report average lactation feed consumption or average daily feed consumption curves [10]. However, factors affecting the variability of the daily feed intake (DFI) pattern throughout lactation have been investigated to a lesser extent.

More than 25 years ago, Koketsu et al. [11] classified the feed intake during lactation of more than 25,000 sows on 30 commercial farms into six feeding behavior patterns. This research showed that sows with a lower total feed intake or with a significant drop during the lactation period had a longer weaning-to-first service interval and lighter weaning litter weights. But these consequences were determined *a posteriori*, i.e., could not be solved.

To prevent these problems, Koketsu et al. [11] proposed that the use of a feed card for each lactating sow was useful to identify sows that eat poorly, to determine the proportion of sows with undesirable feed intake patterns on a specific farm, and to focus attention on the causes of this suboptimal feed intake.

However, in recent years the novel concept of “precision livestock farming” has been developed. It is based not only on the automatic monitoring of livestock via smart sensors or robots, but also on the connection with the related physiological processes.

In this framework, up until now, there were many electronic feeding systems for lactating sows which permits the caregiver to decide and adjust the amount of feed given to every single sow. Nonetheless, new options have arrived on the market recently, that enable the sows not only to eat freely (in quantity) but also to distribute their feed throughout the day according to their preferences. This fact directly affects their feeling of satiety and well-being, but it also allows farmers both to know the lactation pattern and reduces feed wastage. Hence, following the sow’s consumption in real-time allows one to anticipate future production problems that trigger nutrient deficiency, which clearly has consequences at a productive level. More recently, Cabezón et al. [13] investigated lactation sows’ feed intake patterns using mixed models based on polynomial prediction functions for daily consumption. The analysis allowed sorting the lactation records into three groups that showed similar DFI during the first two weeks of lactation but had substantial differences after 18 days. The authors concluded that the substantial differences in DFI suggest high variation in the individual sow’s nutrient requirements and heat production.

This work aimed to characterize the feed intake behavior of modern genetic multiparous lactating sows using electronic feeders and to evaluate its implication in their reproductive outcomes.

## Materials and methods

### Animals and housing

Daily lactation feed intake records were collected on a commercial farm (Centro Experimental Porcino) in Aguilafuente, Segovia, Spain. Sows were housed in an environmentally controlled building with farrowing crates. A total of 1,058 daily feed intake records were collected from 585 Topigs Norsvin TN-70 multiparous sows. The mean lactation length was 27.15 ± 1.13 days. A cycle was defined as the number of reproductive cycles completed from service to weaning, whereas parity was defined as number of times a sow farrowed in its lifetime. Therefore, the data included 229 parity 2656 parity 3–5 and 173 parity 6 or more (parity 6+) sows. Primiparous sows were discarded from the present study because they have different nutrient requirements and feed consumption capacity [14], then their feed pattern might be affected. Nurse sows were excluded from the experiment. During the study all normal management practices were maintained, including cross-fostering. Cross-fostering is the relocation of piglets from their biological mother to a foster sow to equalize litter size and reduce mortality. In this farm, litter size was adjusted within 24 and 48 h after birth, taking into account the functional teats of sows. In addition, throughout the suckling period, runt piglets were removed from the experimental litters to avoid animal suffering. Data were collected using an electronic feeding system (Gestal Solo, JYGA Technologies, Quebec, Canada). The electronic feeding system was programmed in such a way that the sow could have its feed at any time of the day with a maximum of six feedings per day. To be sure that the sow had *ad libitum* feed, but also to reduce feed wastage by playing with the feed, during the first 7 days of lactation the system was used to set an upper limit at 1.20 times the DFI of the previous day. After day 7, DFI was not restricted by an upper limit. In order to unify the data, lactation was set to 28 days and sows with lactation of less than 21 days were removed from the database. The feeders were volumetrically adjusted on and calibrated to the grams for each farrowing group at the entry into the lactation barn of every group of sows.

### Data processing and clustering techniques

The first approach was made with 1058 observations of sow’s consumption in which their feed intake during lactation was studied. A clustering technique was applied to classify the consumption of lactating sows. In machine learning, clustering belongs to the class of unsupervised learning problems whose objective is to determine how the data are organized without any labelled examples. The objective of clustering is the partition of the dataset into homogeneous groups of data, called clusters, where data points in the same cluster are the more similar to each other and dissimilar to data in other clusters [15, 16].

As commented, the sow’s feed intake data from the first 28 days of lactation were used to homogenize the database. For each sow, feed consumption was considered according to the averages of feed consumption in different subperiods of the lactation phase, giving particular importance to what happened during the first half of lactation: from d 0–3, d 4–6, d 7–10, d 11–14, d 15–21, and d 22–28 of lactation. These six subperiod means were used as variables to classify each sow in the clustering.

First, the variables were normalized as [(x-mean(x))/standard deviation(x)] due to the difference of statistical results among them. It means it was necessary to normalize data because, on the contrary, variables with the highest numerical value would have more weight in the model because their numerical value was greater. The next step was to classify each sow by these six variables using Partitioning Around Medoids (PAM) clustering algorithm. This algorithm is quite similar to k-means, although it has some advantages, such as a more robust method for noise and outliers and an individual ‘model’ for each cluster [17, 18]. This algorithm is based on the search of *k* representative objects, and then it assigns each object to the closest medoid; the main objective is to minimize the sum of dissimilarities between the objects in a cluster and the center of the same medoid. The first step for this classification was to select *k* random points as the medoids. Then, each data point was associated with the closest medoid using the Euclidean distance metric method. After that, the cost function (minimize the sum of dissimilarities) was calculated. Each medoid was named *m*, and each point which was not a medoid was named *p*. If this function was decreasing, the medoid (*m*) and the point (*p*) were swapped, and the cost was recomputed again. When the cost function reached the minimum value, the algorithm remembered this *m* and *p* combination and finished.

As it can be seen, the main difference between PAM and *k*-means algorithm is the use of original points in PAM instead of means like is used in *k*-means. The number of clusters (from 2 to 1,058) was a necessary parameter to consider. For this problem, the number five was chosen as the best number of clusters by silhouette coefficient. Henceforth, clusters will be renamed as patterns; hence, five feed intake patterns were defined. This classification was made using R software (version 4.1.0).

### Pattern definition

Patterns were named with two letters, the first indicated the total amount of feed intake (TFI) during the first week of lactation, and the second indicated the TFI during the rest of lactation. Those letters represent the TFI taking as reference the highest feed intake registered. Percentiles respecting the average of TFI were used. Therefore, sows with high TFI were those with a TFI above percentile 90. Medium TFI was considered in sows whose TFI was between percentile 65 and 90, and low TFI were in those sows with TFI below percentile 65. Hence, definitions are as follows: Pattern 1-HH (high/high): feed intake increased gradually after farrowing, and the peak feed intake occurred around day 14 after farrowing and remained constant after that. The TFI of this pattern was high during the whole lactation, so it was taken as the feed intake of reference for the rest of the patterns. Pattern 2-MH (medium/high): sows presented medium TFI during the first week of lactation but high during the rest. Pattern 3-HM (high/medium): sows started lactation with high TFI but decreased until medium during the rest of lactating time. Pattern 4-MM (medium/medium): the TFI of these sows was medium (between percentile 65 and 90) during the whole lactation. Pattern 5-LL (low/low): the TFI was below percentile 65 during the whole lactation.

After obtaining the characterization of the five feed intake patterns, the population of sows (1,058 sows) were classified into them. Daily and weekly average and total feed intake during lactation were calculated for each feed intake pattern group.

### Sow productive parameters

Some analyses were conducted to determine the impact of feed intake patterns on productive parameters. Productive parameters of the current cycle were analyzed, including prolificacy (total number of piglets, born alive, stillborn, and mummified), preweaning mortality (PWM) and number of weaned piglets. Weaning-to-first service interval (WFSI), farrowing rate and prolificacy of sows in the following cycle were also analyzed.

### Statistical analysis

The normality of all productive parameters was evaluated using the test of Kolmogorov-Smirnov. The test of Levene was used to check the homoscedasticity, in other words, the equal variance of the residuals. Once it was verified that the variables could be defined as an approximation of a normal distribution and variance of the residuals were uniformly distributed, data analyses were conducted by using the generalized linear models (procedure GLIMMIX of SAS, version 9.4; SAS Inst. Inc., Cary, NC). The non-parametric Kruskal-Wallis test (procedure NPAR1WAY of SAS) was used in those variables which did not fit a normal distribution or were not homoscedastic. Moreover, contrast analyses in SAS were performed to compare two pattern groups (i.e., 1-HH and 3-HM) and two pattern groups (2-MH and 4-MM) in order to determine the impact of the first period (0–6 d of lactation) on reproductive performance. Contrast analysis between group 5-LL and the rest of pattern groups was also conducted to evaluate the prolificacy in the following cycle.

## Results

### Feed intake patterns evaluation

**Fig. 1:**
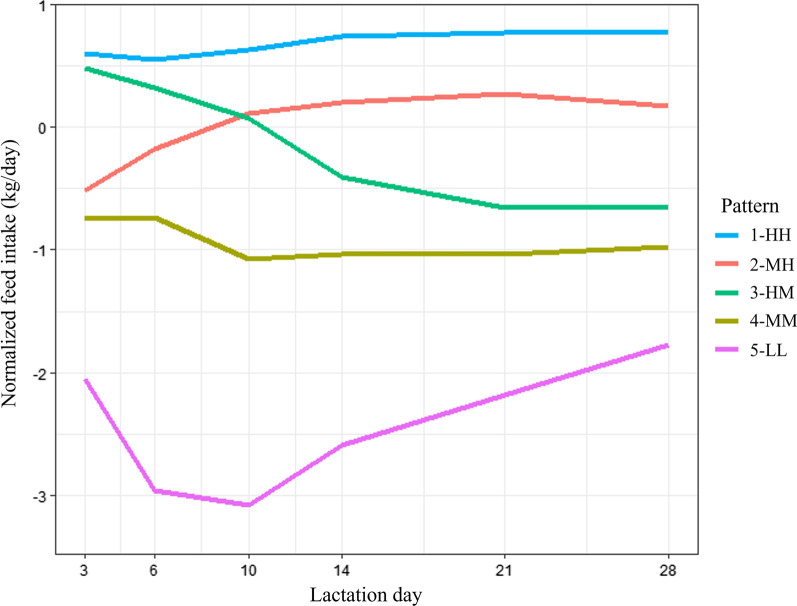
Normalized feed intake patterns of lactating sows. Pattern 1-HH (high feed intake during the first week of lactation/high feed intake during the rest of lactation); Pattern 2-MH (medium/high); Pattern 3-HM (high/medium); Pattern 4-MM (medium/medium); Pattern 5-LL (low/low)

**Fig. 2:**
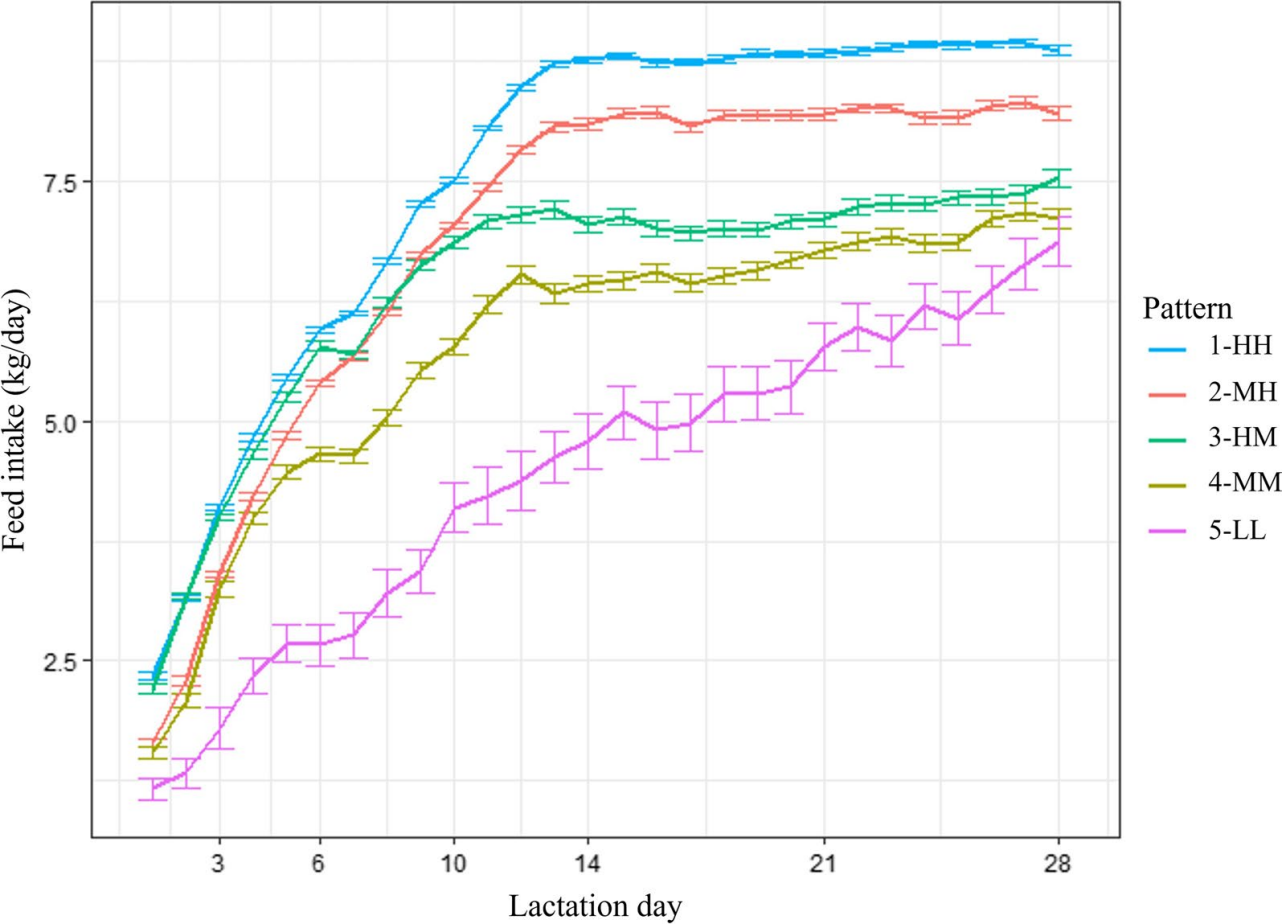
Mean of actual daily feed intake of the different feeding patterns of lactating sows. Pattern 1-HH (high feed intake during the first week of lactation/high feed intake during the rest of lactation); Pattern 2-MH (medium/high); Pattern 3-HM (high/medium); Pattern 4-MM (medium/medium); Pattern 5-LL (low/low)

The first results of the current study were to obtain the feed intake pattern classification per se. Figure 1 shows the normalized feed intake pattern, where it is possible to observe the feed intake behavior of the sows within the different patterns. Figure 2 shows the actual daily feed intake of sows (expressed as mean values) during the lactation period.

Of 1058 observations, proportions of Patterns 1-HH, 2-MH, 3-HM, 4-MM and 5-LL were 38.5, 24.0, 18.4, 15.2 and 3.9%, respectively.

Table 1 shows the TFI considering different periods of lactation. During the first 6 d of lactation Patterns 1-HH and 3-HM had a similar feed intake, followed by Patterns 2-MH, 4-MM and 5-LL. However, as lactation progressed, the patterns become more defined, and similar results were obtained in the different evaluated periods (from 7 to 14 d, 15–21 d and 22–28 d of lactation) in which Patterns 1-HH, 2-MH, 3-HM, 4-MM and 5-LL were ordered by decreasing feed intake. Something similar occurred when considering the TFI of all sows in the whole lactation, which was 167.5 (2.02 SEM) kg, and it decreased as the pattern number increased (*P* < 0.01).

The mean feed intake of all the sows was 6.2 (0.06 SEM) kg across five patterns. As the pattern numbers increased from 1-HH, 2-MH, 3-HM and 4-MM to 5-LL, their mean DFI also decreased from 7.6 to 6.9, 6.4, 5.8, and 4.3 (0.06 SEM) kg, respectively (*P* < 0.01).

**Table 1:** Total amount of feed intake (TFI, kg) during different lactation periods (day 0–6, day 6–14, day 14–21 and day 21–28), whole lactation period and mean daily feed intake (DFI, kg) of sows by five patterns

TFI	Pattern					SEM	*P*-value
	1-HH	2-MH	3-HM	4-MM	5-LL		
No. of sows	407	254	195	161	41		
Sows (%)	38.5	24.0	18.4	15.2	3.88		
0–6 d, kg	25.5^a^	21.6^b^	24.9^a^	19.9^c^	11.9^d^	0.39	< 0.01
7–14 d, kg	61.3^a^	56.8^b^	53.7^c^	46.2^d^	30.9^e^	0.66	< 0.01
15–21 d, kg	61.2^a^	56.8^b^	49.2^c^	45.9^d^	36.0^e^	0.77	< 0.01
22–28 d, kg	57.0^a^	52.6^b^	45.8^c^	43.4^c^	37.0^d^	1.48	< 0.01
0–28 d, kg	204.9^a^	187.8^b^	173.6^c^	155.3^d^	115.8^e^	2.02	< 0.01
DFI, kg	7.56^a^	6.93^b^	6.41^c^	5.75^d^	4.34^e^	0.06	< 0.01

SEM: standard error of the mean

^a-d^Means within a group with different letters are different (*P* < 0.05)

### Association between patterns and reproductive performance

Table 2 shows reproductive performance results obtained in the current reproductive cycle considering the different feed intake patterns. There were no statistical differences in the number of piglets total born, born alive or mummified (*P* > 0.10). However, regarding stillborn piglets and the percentage of stillborn and mummified, the Pattern 5-LL group presented the highest values (*P* = 0.01). Taking into account these findings, a contrast analysis was conducted to determine if there were statistical differences between patterns based on the feed intake start, comparing the two patterns with high feed intake (1-HH and 3-HM) and those with intermediate feed intake (2-MH and 4-MM) during the first week of lactation. The contrast analysis showed that sows with Patterns 1-HH and 3-HM had fewer stillborn piglets and a lower percentage of stillborn piglets and mummies than those with Patterns 2-MH and 4-MM (*P* < 0.01). Preweaning mortality was similar between groups (*P* = 0.20). However, sows with 1-HH had the most pigs weaned, whereas sows with 5-LL had the fewest pigs weaned (*P* < 0.01). Also, sows with Patterns 1-HH and 2-MH tended to have more piglets weaned and lower PWM than those with Patterns 3-HM and 4-MM (*P* **≤** 0.07).

**Table 2:** Reproductive performance (in terms of prolificacy, weaned piglets and preweaning mortality) of lactating sows in the current cycle considering the different feed intake patterns

Period	Pattern					SEM	*P*-value
	1-HH	2-MH	3-HM	4-MM	5-LL		
No. of sows	407	254	195	161	41		
Total born	16.5	16.9	16.4	16.8	16.7	1.14	0.80
Born alive	14.7	14.8	14.7	14.5	13.2	0.72	0.14
Stillborn	1.23^bc^	1.53^bc^	1.07^c^	1.65^b^	2.68^a^	0.55	0.01
Mummified	0.59	0.54	0.66	0.59	0.81	0.19	0.62
%Stillborn + Mummified	10.1^b^	11.3^b^	9.77^b^	13.1^b^	20.3^a^	3.37	< 0.01
Weaned	11.5^a^	11.0^b^	10.9^b^	10.9^b^	9.99^c^	0.71	< 0.01
PWM (%)^1^	15.9	14.9	15.8	15.9	16.9	2.69	0.20

SEM: standard error of the mean

^a-c^Means within a group with different letters are different (*﻿P* < 0.05)

^1^PWM: preweaning mortality

The reproductive performance of lactating sows in the subsequent cycle is presented in Table 3. Feed intake pattern affected the WFSI and farrowing rate, so that sows from Pattern 5-LL tended to have higher WFSI (*P* = 0.06) and had lower farrowing rate (*P* < 0.01) than sows from the other groups. Although there were no statistical differences (*P* > 0.10) in terms of prolificacy in the following cycle (number of piglets total born, born alive, stillborn and mummified). Nonetheless, contrast analysis evidenced that sows with Pattern 5-LL had lower number of born alive piglets (-8.9%; *P* < 0.05) and higher number of stillborn piglets (+ 23.2%; *P* < 0.01) comparing to the rest of patterns.

**Table 3:** Reproductive performance (in terms of weaning-to-first service interval, fertility and prolificacy) of lactating sows in the following cycle considering the different feed intake patterns

Period	Pattern					SEM	*P*-value
	1-HH	2-MH	3-HM	4-MM	5-LL		
No. of sows	407	254	195	161	41		
WFSI (days)^1^	6.34^y^	5.93^y^	6.55^y^	6.50^y^	13.11^x^	1.31	0.06
Farrowing rate (%)	91.0^a^	89.8^a^	89.6^a^	92.6^a^	75.0^b^	–	< 0.01
Total born	17.4	17.7	17.4	19.9	16.3	0.92	0.20
Born alive	14.8	15.1	15.1	14.7	13.6	0.95	0.12
Stillborn	1.89	1.89	1.76	1.67	2.22	0.65	0.22
Mummified	0.68	0.59	0.58	0.53	0.51	0.24	0.63
%Stillborn + Mummified	14.2	13.0	12.9	12.1	15.4	4.15	0.16

SEM: standard error of the mean

^a,b^Means within a group with different letters are different (P < 0.05)

^x,y^Means within a group with different letters tend to be different (*P* = 0.06)

^1^WFSI: weaning-to-first service interval

## Discussion

In the current work, the different consumption patterns of multiparous sows in a commercial farm have been characterized, and their effects on production parameters have been studied.

The patterns obtained in the present study, using a clustering technique, are pretty similar to those proposed in the previous U.S.A. study using 25,000 feed cards of sows fed on 30 commercial farms [11], even though DFI in the present study was 20% or higher (6.2 vs. 5.2 kg/d) than the previous one and parity structures and lactation length differed between two studies.

It is also important to take into account the distribution of sows within these patterns. Twenty-five years ago, more than 50% of sows belonged to minor (25.8%) and major drop (38.3%) patterns [11], while currently 50% of the sows are placed in Patterns 1-HH (38.5%) and 2-MH (24.0%).

Using mixed-effects linear models with the DFI data in Canada, collected from electronic feeders during lactation, three groups of feeding curves were reported by U.S.A. researchers [13]. They concluded that there were no differences in DFI during the first two weeks of lactation between the three groups, but the DFI during 18–28 d of lactation differed between them: decrease (14.1% of sows), increase (18.6% of sows) and minor changes (67.3% of sows). Additionally, our normalized DFI figure shows there were slight differences of DFI during 18–28 d of lactation within the patterns except for 5-LL. These differences might be due to farm differences between North American and European studies such as gestation group housing and lactation crates, genetic lines, parity structures and feed ingredients.

Concerning the differences reported by Cabezón et al. [13] from day 18 onwards, it is worth noting that in the current study, once the peak of lactation is reached (around day 18), it is observed that the patterns show small variations in the slope (see normalized feed intake graph; Fig. 1). It seems that Patterns 1-HH and 3-HM remain the same (representing 56.9% of sows), Pattern 2-MH shows a slight decrease (24.0% of sows), and Pattern 4-MM shows a small increase (15.2% of sows). Observing the results of both studies, it can be concluded that once the lactation peak is reached, the daily sow consumption remains at these values, with very small variations.

As mentioned above, with precision livestock farming and the development of various devices, continuous and automated data collection is possible. In this sense, adapted machine learning techniques, as used in the current work, allows the extraction of valuable knowledge from this data. The most recent research regarding this topic is the one conducted by Gauthier et al. [19], who combined the use of electronic feeders as well as the development of specific computational methods for storing and dealing with time series [20] to provide a different description of feed intake. Gauthier et al. [19] used a time-series clustering technique for trajectory curves with the DFI data during 20 d of lactation on six farms collected from electronic feeders. As a result, they found two clusters or feeding curves and concluded that both curves were similar after 5 d of lactation. A possible reason for the difference between the previous two studies [13, 19] and our results might be that they did not categorize the feeding curves into some patterns by a decrease for individual DFIs of sows. Our results are similar to a previous U.S.A. study which categorized individual DFIs into six patterns using a major drop, a minor drop, a rapid increase, a gradual increase, low DFI only in early lactation and low DFI throughout lactation [11].

In relation to this, Dourmad [21] observed a drop in feed intake at about five days of lactation in lean sows with a high appetite which was *ad libitum* fed from the day of farrowing. These authors suggested that this drop was related to the occurrence of gastrointestinal disorders resulting from uncontrolled excessive feed intake at the beginning of lactation.

However, in the present study, some variations, including small drops, were present in most of patterns. It can be attributed to the variation of the individual feed intake in each day of lactation and also in the fact of using electronic feeders which makes it possible to limit the risk of overconsumption and provide better control of individual needs and, in consequence, a decrease in the frequency of major drops in patterns.

It is worth mentioning that if previous studies are reviewed, feed intake during lactation has increased in the last 30 years by around 29% (between 21 and 44%) [11, 13, 19], confirming the increase of sow productivity. Moreover, in all studies, it seems that the largest differences in feed intake patterns are found towards the end of lactation, probably due to the large variability in appetite between individual animals in successive days [22].

The absence of pain and the well-being of the sow after farrowing is a direct determinant of their intake during lactation, especially at the beginning. In this sense, dystocia can be defined as a parturition difficulty resulting from prolonged spontaneous farrowing and is associated with unacceptably high levels of pain [23]. The duration of farrowing is normally linked with an increase in the number of stillborn piglets [24, 25]. Several authors establish the percentage of stillborn piglets between 3 and 12%, in accordance with those from this work (between 6.5 and 9.8%), except sows characterized as Pattern 5-LL. These sows presented 16.3% of stillborn piglets, which may have resulted in a more painful farrowing and, in consequence, lower intake throughout the lactation.

However, the consequence of farrowing was not only reflected in the Pattern 5-LL. The results of this work showed that the sows characterized as Patterns 1-HH and 3-HM had a better intake start during lactation than those from Patterns 2-MH and 4-MM. This could be because sows from Patterns 1-HH and 3-HM had a lower percentage of stillborn and mummified piglets than sows from Patterns 2-MH and 4-MM (9.95% vs 12.2%, respectively). This could imply a less painful farrowing and, therefore, a higher intake in the first days of lactation.

Although there were no differences between feed intake patterns in the number of piglets total born and born alive nor in PWM, it is worth noting that sows from Pattern 5-LL had the lowest number of weaned piglets. This could be due to cross-fostering practices conducted by caretakers as normal management on a commercial farm. Although ideally, fostering should take place 12–24 h after birth, it is well-recognized that fostering piglets is time-sensitive and can be done throughout the lactation to minimize mortality [26, 27].

Previous researchers revealed that light birth weight piglets reared in uniform litters had heavier weaning weights and fewer removal than those reared in mixed litters, using a cross-fostering technique with light and heavy birth weight piglets [28]. This evidence aligns with the results of the current study, where the lower number of weaned piglets found in sows with Pattern 5-LL could be due to the removal of runt piglets to a foster sow with lighter piglets.

Postweaning reproductive performance is affected by the extent and timing of catabolic losses of maternal tissues during lactation due to being prioritized towards milk production [29]. Also, milk production is suddenly interrupted after reaching the peak, and they are separated from piglets and moved to a different place [30].

The change from lactational anestrus to the cyclic phase occurs in very few days, 4–6, on commercial farms [31, 32], which can explain a few days differences in WFSI between Patterns 1-HH, 2-MH, 3-HM and 4-MM. Sows with Pattern 5-LL might be highly catabolic due to low DFI throughout lactation which is similar to those with low DFI assigned during three weeks and having prolonged WFSI in the study of Koketsu [33].

Additionally, low LH secretion during lactation is a key connection from low DFI and nutritional status to postweaning reproductive performance of sows [33–35]. In our research, sows with Pattern 5-LL have prolonged WFSI and have a lower farrowing rate than those with other patterns.

## Conclusion

Our study indicates the importance of reaching Pattern 1-HH by having a rapid increase and a stable plateau in feed intake during lactation, which is associated with high weaning performance and subsequent reproductive performance of the sows. Also, our study suggests that Pattern 1-HH is linked to a good farrowing with a low percentage of stillborn piglets and mummies. On the other hand, sows with other patterns might have problems associated. In particular, our pattern categorization found 3.9% sows with 5-LL pattern, low feed intake throughout lactation. Finally, it is critical for producers and veterinarians to identify a problematic sow on time to make a prompt intervention decision.

## Data Availability

The dataset used and/or analysed during the current study are available from the corresponding author on request.

## Abbreviations

DFI: Daily feed intake
LH: Luteinizing hormone
PAM: Partitioning around medoids
PWM: Preweaning mortality
TFI: Total amount of feed intake
WFSI: Weaning-to-first service interval
